# A comparative investigator-initiated pilot study on the efficacy and safety of biodegradable microneedle acupuncture and conventional intradermal acupuncture for dry eye patient: A superiority, assessor-blinded, randomized controlled trial

**DOI:** 10.1097/MD.0000000000031468

**Published:** 2022-11-25

**Authors:** Ji-Hoon Song, Soo-Yeon Park

**Affiliations:** a Department of Ophthalmology, Otolaryngology & Dermatology, College of Korean Medicine, Dongshin University, Naju-si, Republic of Korea.

**Keywords:** biodegradable microneedle acupuncture, dry eye, intradermal acupuncture, protocol, randomized controlled trial, thumbtack needle

## Abstract

The prevalence of dry eye, which is a common lacrimal disease, is steadily increasing in modern society. However, fundamental treatment for it has not yet been established. This study aims to assess the efficacy and safety of a novel medical device, the biodegradable microneedle acupuncture (BMA), using a traditional intradermal acupuncture needle as the control acupuncture for dry eye.

**Method:**

This study will be an investigator-initiated, assessor-blinded, comparative, superiority pilot randomized controlled trial. A total of 30 patients with dry eye will be randomly assigned to the experimental or the control group in equal proportion. For the experimental group, the BMA will be applied to both sides of 5 acupoints including BL2, GB14, TE23, EX-HN5, and ST1. For the control group, conventional intradermal acupuncture will be applied to the same acupoints. The needles will be attached for 4 hours. Over 4 weeks, both the interventions will be performed 12 times in total. The primary outcome would be the ocular surface disease index. The secondary outcomes would be the visual analog scale for subjective symptoms, quality of life, Schirmer I test, and general assessment.

**Discussion:**

The findings of this study on the efficacy and safety of the BMA would be helpful for patients with dry eye in clinical practice. Further, these results would provide for the foundation of a large-scale BMA study.

## 1. Introduction

Dry eye syndrome is a lacrimal disease caused by insufficient secretion or excessive evaporation of tears, which leads to ocular surface damage, and accompanies irritating symptoms, including discomfort, foreign body sensation, or dryness of the patient’s eye.^[1]^ According to a report from the National Health Insurance Service of Korea in 2018, the prevalence of dry eye has steadily increased by 2.1% annually since the recent 5 years.^[2]^ Nowadays, dry eye can be aggravated by various environmental factors, which include excessive use of visual display terminals, increase in indoor stay, fine dust, or growing use of contact lenses.^[3–5]^ Although artificial tears, corticosteroid eye drops, cyclosporine, therapeutic contact lenses, protective glasses, and punctal occlusion have been suggested as treatments, they have several side effects and are not considered fundamental treatment options.^[6–9]^

In Korean medicine, acupuncture or herbal medication, which clear heat, nourish the yin and body fluid, or supplement the blood and qi, have been traditionally used for dry eye.^[10]^ Clinically, acupuncture has been considered the main Korean medicine treatment for dry eye. Hence, studies^[11–14]^ on the efficacy of acupuncture for dry eye have been steadily reported; however, these prior studies are mainly limited to case reports. Thus, a randomized controlled trial (RCT) is necessary to establish the evidence of acupuncture for dry eye.

Intradermal acupuncture (IDA) is a type of acupuncture commonly used for dry eye in clinical practice; however, related clinical studies are rare. Thus, we conducted preliminary studies on the efficacy and safety of IDA for dry eye. First, we searched RCTs on IDA for dry eye from domestic and foreign databases, and analyzed 4 eligible RCTs.^[15]^ Thereafter, we conducted a preliminary RCT (not published yet) comparing IDA with body acupuncture for dry eye. This study has suggested significant improvement of the ocular surface disease index (OSDI) in both interventions, no difference upon intergroup comparison, and safety of IDA.

This study assesses the efficacy and safety of a biodegradable microneedle acupuncture (BMA) for patients with dry eye compared to conventional IDA. Based on the results, this study aims to demonstrate the superiority of BMA for dry eye compared to conventional IDA.

## 2. Method

### 2.1. Study design

This study is designed as an investigator-initiated, parallel, superiority RCT. Patients with dry eye who voluntarily agree to this study would be screened using the necessary examinations and eligibility criteria. The suitable participant would be randomly allocated to the experimental group or the control group on visit 1. The participants allocated to the experimental group would be treated with the BMA. In contrast, the participants allocated to the control group would be treated with IDA. For 4 weeks, both the groups would be treated 3 times a week (total 12 times) by each intervention. Within 4 weeks from visit 1, the investigators would conduct the final assessment after treatment termination, and the trial would be completed.

### 2.2. Sample size and recruitment

The exact calculation of the number of participants for statistical analysis for this pilot study is challenging because owing to the few reports on BMA. The number of participants for a pilot study should be at least twelve people per group as suggested by a previous study.^[16]^ Considering a dropout rate of 20% owing to a total of 12 treatments for 4 weeks, this study requires fifteen participants per group. Thus, the total required participant number for this study is thirty.

Participants would be recruited via advertisements in a single hospital. If a patient desires to participate, investigators would detail the study. Then, the participant who voluntarily submits the written consent would be screened using the inclusion and exclusion criteria. If considered suitable, the participant would receive a unique participant number.

### 2.3. Participants

#### 2.3.1. *Inclusion criteria*.

Participants should satisfy all the following criteria:

1. Male and female aged between 19 and 65 years.
2. Patients with dry eye having typical symptoms, including pruritus, foreign body sensation, irritation, pain, dryness, blurry eye, dazzle, eye congestion, and excessive lacrimation, and whose OSDI score is more than 13.
3. Less than 10 mm/5 min in the Schirmer I test.
4. Those who voluntarily submit the written consent.

#### 2.3.2. *Exclusion criteria*.

Patients who satisfy any criterion of the followings would not be able to participate:

1. Dry eye symptoms attributed to the eyelid or eyelashes.
2. Eye irritation attributed to acute inflammation of the eyelid, eyeball, or ocular appendage.
3. Skin diseases including Stevens–Johnson syndrome or pemphigoid.
4. Vitamin A deficiency.
5. Eyeball or ocular appendage injuries.
6. Received operations via anterior chamber, including cataract or laser-assisted in situ keratomileusis, within recent 3 months.
7. Having a difficulty in blinking owing to facial palsy.
8. Received punctal plug or punctal occlusion.
9. Used anti-inflammatory eye drops including cyclosporine, corticosteroids, or autologous serum eye drops recently within 2 weeks.
10. Active ocular infections, including anterior uveitis or anterior blepharitis.
11. Those who have ocular allergy or are undergoing treatments for allergic eye diseases.
12. Those who have symptoms or are undergoing treatments for viral conjunctivitis.
13. High intraocular pressure of more than 25 mm Hg or glaucoma of any eye.
14. Taking systemic corticosteroids or immunosuppressants treatment.
15. Pregnant, nursing women, or women planning pregnancy.
16. Others considered ineligible by the investigators.

### 2.4. *Randomization and allocation concealment*

Block randomization will be used to divide the participants into 2 groups. The randomization will be conducted by a statistical software (SAS; SAS Institute Inc., Cary, NC). Setting the block size as 4, total 30 people would be randomly allocated into 2 groups with the same possibility. Thus, 15 participants would be assigned to each group.

Allocation would be decided after screening. Investigators will open an opaque and sealed envelope, which contains the allocation result, in front of the suitable participant. The opened envelope will be stored separately.

### 2.5. Blinding

This study is single-blind, which only blinds the assessor. Double-blinding is challenging for this study since the participants and investigators would be aware of the applied treatment. Moreover, the investigators who treat interventions cannot objectively assess the result. Thus, the assessor would be blinded to strengthen the objectivity of this study and reduce the bias of the investigators.

In order to ensure blinding, the roles of the investigators would be categorized into the assessor, clinical research coordinator, and therapist. The assessor would not be able to contact the participants except for the assessment time. An additional assessment form, which does not contain identifiable personal information, would be prepared for the assessor.

### 2.6. Interventions

#### 2.6.1. *The experimental group*.

**Figure 1.**
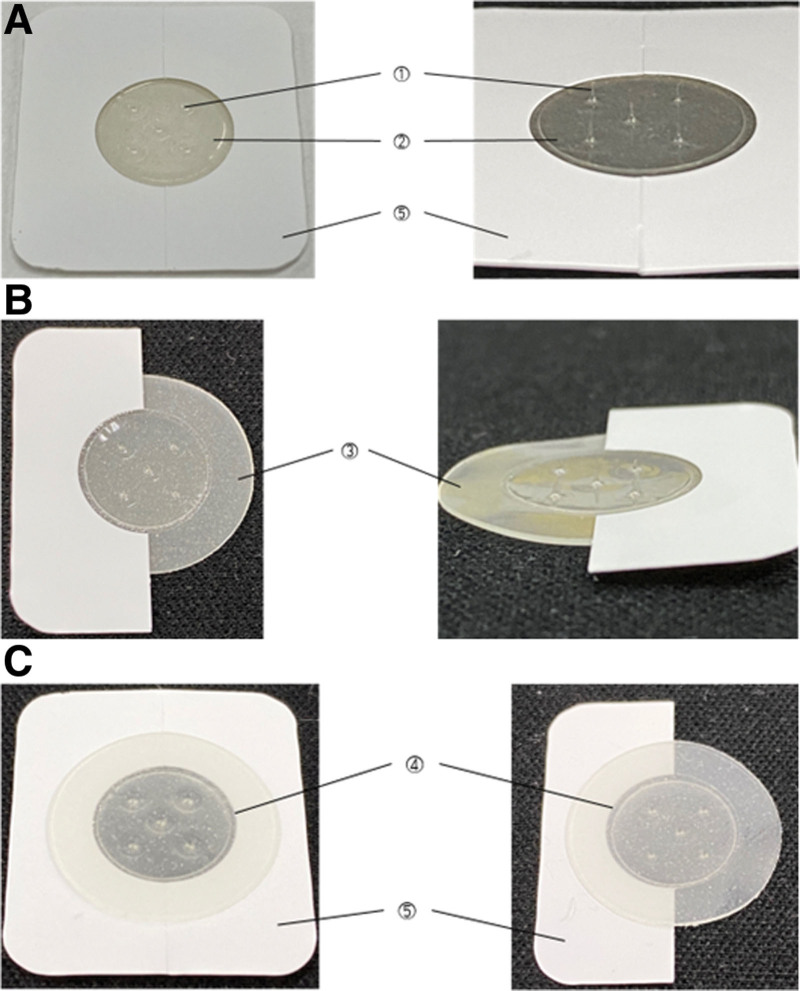
Structure of BMA. (A and B) Anterior side of the BMA. (C) Posterior side. The numbers in the figures indicate each component of the BMA; ①: microneedles, which are inserted into the skin; ②–④ constitute a sheet of substrate film; ②: microneedle-supporting film; ③: adhesive surface, which serves as adhesion to the skin; ④: adhesive surface protective film, which covers the posterior side of the adhesives; ⑤: release paper, which protects the adhesive surface and is separated before use. BMA = biodegradable microneedle acupuncture.

The experimental group would be treated using a disposable sterilized BMA (RMD-PN1; Raphas Co. Ltd., Seoul, Republic of Korea). The basic shape of BMA is the same as a microneedle patch. Five biodegradable hyaluronic acid microneedles are attached to a hydrocolloid film (Fig. 1). The specifications of the BMA are presented in Table 1. BMA would be applied to both sides of 5 acupoints, including BL2, GB14, TE23, EX-HN5, and ST1, by Korean medicine specialists or residents who have more than 3 years of clinical experience. Attached BMAs would be removed after 4 hours. For 4 weeks, the participants would receive the treatment 12 times in total.

**Table 1:** Specifications of biodegradable microneedle acupuncture.

Model name	RMD-PN1
The length of needle (mm)	1.2
The thickness of needle (μm)	<80
Array spacing (mm)	>1.4
Total length (mm)	
Width	20
Length	24
Substrate film length (mm)	
Width	16
Length	16
Weight (mg)	124 ± 12

#### 2.6.2. *The control group*.

**Figure 2.**
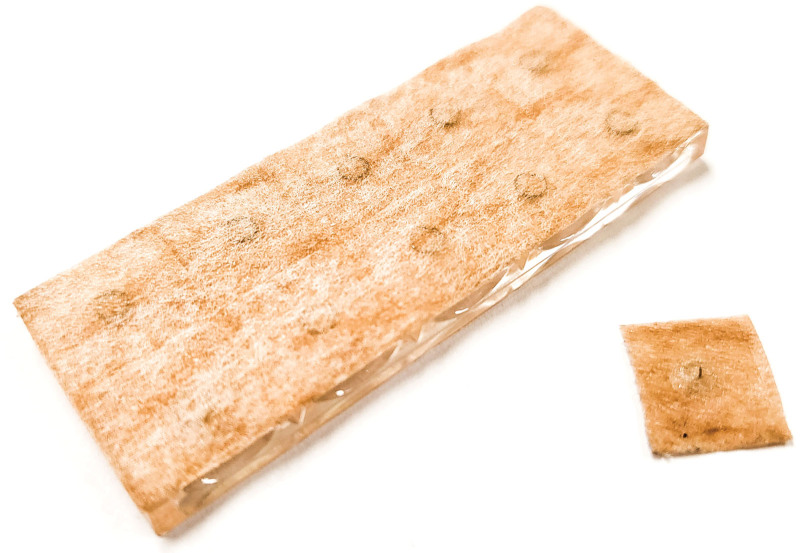
Conventional intradermal acupuncture needle. A small sterilized stainless steel thumbtack needle fixed on a square adhesive plaster. Ten pieces of needle are bundled in a transparent plastic baseplate. When using, take a piece off from the baseplate and attach it to the targeted acupoint.

Considering the intervention for the control group, IDA would be used with a disposable sterilized IDA needle (T-press needle DB130A; Dongbang Medical, Seongnam, Republic of Korea), also called “thumbtack needle.” A 0.18 mm × 1.0 mm-sized stainless-steel needle is attached to a small piece of paper plaster (Fig. 2). IDA would be applied to both sides of 5 acupoints, including BL2, GB14, TE23, EX-HN5, and ST1, by Korean medicine specialists or residents who have more than 3 years of clinical experience. Attached IDA needles would be removed after 4 hours. For 4 weeks, the participants would receive the treatment 12 times in total.

### 2.7. *Prohibited and permitted concomitant treatment*

During the trial, all kinds of concomitant treatments, including Korean medical therapy, would not permitted to both groups. The concomitant use of medications including anti-inflammatory eye drops, corticosteroids, or immunosuppressants would be prohibited. Participants who used such unauthorized medications would be dropped out from the study, and excluded from the final analysis.

### 2.8. Study procedure

Participants would visit a total 14 times for this study. The visits consist of screening, treatment and follow-up (visit 1-12), and final follow-up (visit 13) visits. The overall study schedule is presented in Table 2.

**Table 2:** Study schedule and checklist for each visit.

Phase	Screening*	Treatment and follow-up												
Visit		1*	2	3	4	5	6	7	8	9	10	11	12*	13^†^*
Time		Week 1			Week 2			Week 3			Week 4			
Informed consent	●													
Sociodemographic information	●													
Medical and medication history	●	●	●	●	●	●	●	●	●	●	●	●	●	●
Physical examinations	●													
Eligibility criteria	●													
Vital signs	●	●	●	●	●	●	●	●	●	●	●	●	●	●
Randomization		●												
BMA or IDA treatment		●	●	●	●	●	●	●	●	●	●	●	●	
Visual acuity test	●	●	●	●	●	●	●	●	●	●	●	●	●	●
OSDI	●	●						●						●
VAS for subjective symptoms		●						●						●
Schirmer Ⅰ test	●	●												●
General assessment														●
Quality of life		●						●						●
Adverse events		●	●	●	●	●	●	●	●	●	●	●	●	●
Compliance assessment														●

BMA = biodegradable microneedle acupuncture, IDA = intradermal acupuncture, OSDI = ocular surface disease index, VAS = visual analog scale.

* The visits allowed to schedule on the same day = screening visit and visit 1, visit 12 and 13.

† Visit 13 is scheduled within 3 days from the time when 4 weeks passed from the visit 1.

#### 2.8.1. *Screening visit*.

Before trial initiation, patients who want to participate in the study would be thoroughly explained about the procedure by the investigators, and the participants would be requested to voluntarily submit the written consent. Then, interview and physical examination would be conducted to assess the eligibility. Collected data are the demographic and sociological information, medical history, treatment history, vital signs (blood pressure, pulse rate, and body temperature), visual acuity tested by Dr Hahn’s vision Test Chart for 3 m, and intraocular pressure measured by a tonometer (HNT-1; Huvitz Co., Ltd., Anyang, Republic of Korea). In accordance with the inclusion and exclusion criteria, suitable participants will receive an identification code, and the eligibility judgement would be recorded on the case report form (CRF). For the selected participants, the next visit date would be designated.

#### 2.8.2. *Visit 1*.

On visit 1, randomization and initial treatment would be executed. Visit 1 is scheduled within 7 days after the screening visit. Participants who passed screening are allowed to receive examinations and treatment of visit 1 on the same day as the screening visit. During this visit, investigators would assess the changes in medical and medication history of the participants, and record them on the CRF. Visual acuity, visual analog scale (VAS) for subjective symptoms, OSDI, Schirmer I test, and quality of life (QoL) will also be examined. Certain examinations, including vital signs, changes in the medical and medication history, visual acuity, and adverse events (AEs) will be conducted in every visit. Before treatment, the participants would be randomly allocated to the experimental or control group. The therapist would treat the participants with the BMA or the IDA according to the allocation result. At the end of the visit, the clinical research coordinator would schedule the next visit.

#### 2.8.3. *Visits 2 to 12*.

From visit 2 to 12, investigators would record the changes in regular examinations, including visual acuity, medical and medication history, and AEs, on the CRF since the last visit. The allocated intervention would be continuously performed for the participant during this period. At the end of each visit, the next visit would be scheduled. Midterm assessment for some outcomes would be executed on visit 7. Before treatment during the visit, the VAS for subjective symptoms, the OSDI, and the QoL would be examined.

#### 2.8.4. *Visit 13*.

Visit 13 is the final follow-up visit, and would be scheduled within 3 days from the time when 4 weeks passed from the visit 1. Investigators would record the changes in the routine examinations since visit 12 on the CRF. The final assessment would be performed for the primary and secondary outcomes, including the OSDI, the VAS for subjective symptoms, the QoL, the Schirmer I test, and general assessment by the participants and investigators. Participants’ compliance for this trial would be also examined during this visit.

#### 2.8.5. *Extra visits*.

Extra visits can be scheduled if the participants request for them or based on the investigators’ decision considering its necessity. It is allowed only if the AEs persist until the end or suspension of the trial, or if there are demands for extra follow-ups after treatment termination from the investigators or the participants. If permitted, the investigators would carry out follow-ups for the emerged AEs.

### 2.9. *Outcomes*

#### 2.9.1. *Primary outcome*.

The primary outcome of this study is OSDI.^[17]^ The OSDI is assessed on visit 1 (prior to intervention), 7, and 13. The OSDI consists of total 12 questions for visual acuity (6 questions), ocular symptoms (3 questions), and provoking environmental factors (3 questions).^[18]^ The severity is scored as none of the time = 0, some of the time = 1, half of the time = 2, most of the time = 3, and all of the time = 4. The total score is calculated according to the following formula.^[19]^


$$\begin{array}{ll} & OSDI\;score\\ & \;\;\;=\frac{(sum\;of\;scores\;for\;all\;questions\;answered)\times 100}{(total\;number\;of\;questions\;answered)\times 4} \end{array}$$

If a respondent does not correspond to a question, the question is not scored and it is treated as “not applicable.” The range of an OSDI score is from 0 to 100, and a higher score indicates more severe symptoms. The values of 0–12, 13–22, 23–32, and 33–100 indicate normal, mild, moderate, and severe dry eye, respectively.^[18,20]^

#### 2.9.2. *Secondary outcomes*.

##### 2.9.2.1. *VAS for subjective symptoms*.

The VAS is evaluated by marking the overall severity of discomfort arisen from dry eye during the last week on a 100 mm straight line. The left end (0 mm) indicates “no symptoms,” and the right end (100 mm) indicates “the worst discomfort.” When the participants mark a dot on the VAS line, investigators score it by measuring the length from the left end to the dot. The score ranges from 0 to 100. The measurement is conducted on visits 1, 7, and 13.

##### 2.9.2.2. *Quality of life*.

The QoL assesses the life of the participants associated with dry eye symptoms during the last week. It is scored from 0 (the worst) to 6 (the best) by the participants themselves. The following is a question to the participants: “During the last week, how much was the overall quality of your life related to dry eye symptoms?” The assessment is conducted during visits 1, 7, and 13.

##### 2.9.2.3. *Schirmer I test*.

The Schirmer I test is used to measure the tear secretion. Upon folding the tip, the Schirmer strip (BIO Color Tear Test; Bio Optics, Seongnam, Republic of Korea) is inserted to the lateral 1/3 point of the lower eyelid. The folded tip should be in the conjunctival sac. Facing their eyes up, the participants close their eyes for 5 minutes immediately after inserting the strips. After 5 minutes, the investigators measure the length of the strip soaked in tears. The test is conducted during the screening visit and visits 1 and 13.

##### 2.9.2.4. *General assessment*.

The general assessment in this study indicates a comprehensive assessment for the improvement in dry eye symptoms using a 5-grade Likert scale.^[21]^ It is assessed by the participants themselves or investigators during visit 13, the final visit upon termination of all the treatments. The responses to the questions include excellent, good, fair, poor and aggravation. “Excellent” corresponds to the responses that dry eye and its concomitant symptoms were completely improved, the treatment demonstrated clear therapeutic effect, or daily life activities were obviously improved. “Good” corresponds to the response that dry eye and its concomitant symptoms still remain; however, they have greatly improved compared to before participating in this trial. “Fair” corresponds to the response that dry eye and its concomitant symptoms remain; however, they are slightly improved or tend to improve after the trial. “Same” corresponds to the response that little change is observed comparing before and after the trial. “Aggravation” corresponds to the response that dry eye symptoms are more severe than before participating in this trial.

#### 2.9.3. *Safety assessment*.

AE includes any unintended results that occurred even though the medical device was appropriately used according to the instructions. The expected AEs of the IDA are local pain from the treated area, local bleeding from the treated area, allergic reactions of the skin, infections, localized infection symptoms (redness, swelling, and/or local pain), nausea, vomiting, headache, and/or dizziness.^[22,23]^ For the BMA, redness on the treated area, skin irritation, and/or skin erosion are also expected in addition to the expected AEs of the IDA. Investigators would check and record the emergence of AEs on the CRF at each visit.

### 2.10. *Statistical analysis*

#### 2.10.1. *Analysis for the primary and secondary outcomes*.

The same analysis method will be used for the primary and secondary outcomes. The measurements from each group would be presented as descriptive statistics, including mean, median, standard deviation, minimum, and maximum. In the intragroup comparisons, the differences in the outcomes following the interventions would be analyzed using the Student’s paired *t* test or the Wilcoxon signed-rank test, and 95% confidence intervals would be presented. The differences between the 2 groups on each visit would be statistically assessed using the 2-sample *t* test or the Mann–Whitney test, depending on whether the normality is satisfied. For missing values, a last observation carried forward method would be used to handle.

#### 2.10.2. *Analysis for safety*.

Any emerged AEs in this study would be listed with detailed descriptions. The frequency of relevant and irrelevant AEs to the interventions would be recorded. The Fisher’s exact test would be used to analyze whether the incidence of reported AEs is different between the 2 groups. In addition, descriptive analysis would be conducted for the severity and type of AEs to determine the difference between the 2 groups.

### 2.11. *Suspension and dropout*

#### 2.11.1. *Criteria for discontinuing*.

1. Violating inclusion criteria or satisfying exclusion criteria.
2. Emergence of serious AEs, or challenging in trial continuation due to AEs.
3. A participant or his/her legal representative demands for discontinuing the trial owing to unsatisfactory therapeutic effect.
4. Compliance with the trial is below 80%.
5. Violations of the study protocol by investigators or participants.
6. Withdrawal of participant’s agreement to the study.
7. Loss to follow-up.
8. During the treatment visits or the follow-up visit, the medications which can influence on the outcomes assessment were used without the investigators’ permission.
9. Others considered inappropriate to proceed the trial by the investigators.

#### 2.11.2. *Criteria for exclusion from analysis*.

If a severe protocol violation occurs, the participant would be excluded from the final analysis, and the data would be analyzed using an intention-to-treat analysis. The followings are severe violations of the protocol:

1. Violations of the eligibility criteria.
2. Used prohibited concomitant medications during the trial.
3. The assessment for primary outcome was omitted at the beginning and/or end of the trial.
4. The number of treatment did not exceed 80% of total scheduled treatments.

For minor violations, investigators would accurately record the degree of violation and its reason. Thereafter, an investigator, a statistician, and a monitor would comprehensively consider the influence on the trial when writing the outcome report. The data would be analyzed using a per-protocol analysis.

### 2.12. *Ethics and dissemination*

This study and related documents, including the study protocol, CRF, informed consent, were approved by the Institutional Review Board of Naju Dongshin University Korean Medicine Hospital (Approval No. NJ-IRB-015). This study was registered with the Clinical Research Information Service, Republic of Korea (Registration No. KCT0007358). The tenets of the Declaration of Helsinki would be abided by investigators while conducting the trial. Prior to beginning the study, the investigators would fully inform the participants of the objective of this study and provide detailed information, and receive written consents from them. The results from this study would be published in a peer-reviewed international journal.

### 2.13. Trial status

Ongoing. This study has begun with the first participant enrollment on July 6th, 2022.

## 3. Discussion

Many studies have introduced the superior therapeutic effect of acupuncture for dry eye than artificial teardrop.^[14,24]^ Furthermore, we concluded that IDA is effective for dry eye based on the RCTs that compared IDA and artificial teardrop in our prior literature review.^[15]^ BMA, a newly developed medical device, originated from the traditional IDA needle but resolved its shortcomings. Thus, this study aims to assess the superiority of BMA to the conventional IDA for dry eye by conducting a clinical trial.

IDA is a type of acupuncture that embeds a small needle in the skin for a long time, to stimulate the acupoints with prolonged weak stimulus. This therapy combined “shallow needling” among ancient needling techniques with “needle retention method,” which was described in the “Huangdi’s Internal Classic.”^[25]^ IDA has lesser pain than conventional body acupuncture, and it does not interrupt daily life or physical activity even though the needle is attached to the patient’s body. Using these features, many Korean medicine doctors have used IDA to treat diseases that require prolonged needle retention to the body surface, including obesity, ceasing smoking, nervous headache, stomachache, migraine, eye strain, dry eye, hypertension, asthma, or menstrual irregularity.^[26–29]^ However, the IDA needle can result in foreign body sensation to the patient if attached for too long, or provoke metal allergic symptoms, including skin inflammation and pruritus, owing to its material.

To overcome the limitation of the IDA needle, BMA used in this study was developed by the Raphas Co. Ltd. The material for the microneedle was changed from stainless steel to biodegradable macromolecule, hyaluronic acid. The safety of hyaluronic acid has been proved by its various applications to eye drops, dermal fillers, and moisturizer in cosmetics. The microneedles of the BMA made from hyaluronic acid completely decompose when inserted to the skin; therefore, they do not provoke allergic reactions in the skin compared to the metal needle.^[30]^ In addition, BMA minimized the risk of infection by gamma sterilization.

BMA is based on a microneedle patch. Originally, the microneedle patch was developed in 1976 to overcome the low efficiency of transdermal drug delivery.^[31]^ It enhances drug absorption into the skin by penetrating the skin barrier with attached microneedles.^[32]^ Owing to these features, the microneedle patch has been widely applied to various fields, including pharmaceuticals, cosmetics, dermatology, and vaccination.^[30,33–38]^ Meanwhile, BMA looks similar to the conventional IDA needle upon observing their appearance. Except for the number of needles and their material, the appearance of both acupuncture needles is fundamentally the same consisting of a small needle with a needle-fixing film. Thus, the BMA can be expected to have similar therapeutic effects of acupuncture as the IDA needle, in addition to the original microneedle patch.

This study has some limitations. First, double blinding is impossible because participants can comprehend the intervention they receive based on the treated needle’s external difference. BMA has a circular translucent film, while the IDA needle has a square apricot plaster. Instead, we blinded the assessor. An investigator in charge of the outcomes assessment would be separated from contacting with the participants except for the assessment time. Moreover, a separate assessment form that cannot verify the participant’s identity is served to the assessor. Another limitation arises from the small sample size. This study recruits fifteen participants for each group. Thus, we may be unable to get the intended results. However, since this study was designed as a pilot trial, we are planning to conduct a further large-scale confirmatory clinical trial based on the results of this study.

In conclusion, the superiority of BMA possibly proven by this study could suggest new potential treatments for dry eye and the application of a microneedle patch. Furthermore, the results from this study will also serve as evidence for further large-scale BMA studies.

## Author contributions

**Conceptualization:** Soo-Yeon Park.

**Funding acquisition:** Soo-Yeon Park.

**Investigation:** Ji-Hoon Song, Soo-Yeon Park.

**Methodology:** Soo-Yeon Park.

**Project administration:** Soo-Yeon Park.

**Supervision:** Soo-Yeon Park.

**Visualization:** Ji-Hoon Song.

**Writing – original draft:** Ji-Hoon Song.

**Writing – review & editing:** Ji-Hoon Song, Soo-Yeon Park.
